# Contraceptive Use Among Women at Risk for Unintended Pregnancy in the Context of Public Health Emergencies — United States, 2016

**DOI:** 10.15585/mmwr.mm6732a6

**Published:** 2018-08-17

**Authors:** Karen Pazol, Sascha R. Ellington, Anna C. Fulton, Lauren B. Zapata, Sheree L. Boulet, Marion E. Rice, Shanna Cox, Lisa Romero, Eva Lathrop, Stacey Hurst, Charlan D. Kroelinger, Howard Goldberg, Carrie K. Shapiro-Mendoza, Regina M. Simeone, Lee Warner, Dana M. Meaney-Delman, Wanda D. Barfield, Behavioral Risk Factor Surveillance System Family Planning Module Working Group, Behavioral Risk Factor Surveillance System Family Planning Module Working Group, Leah Atwell, Rana Bayakly, Sarah Conklin, Harley T. Davis, Victoria Davis, Laurie Freyder, Christine Graham, Sarojini Kanotra, Sarah Khalidi, Georgette Lavetsky, Kristi Pier, Lauren Porter, Mina Qobadi, Sondra Reese, Chelsea L. Richard, Nagi Salem, Ruby A. Serrano-Rodríguez, Birgit A. Shanholtzer, Holly Sobotka, Amilcar Soto-Mercado, Carol L. Stone

**Affiliations:** ^1^Division of Reproductive Health, National Center for Chronic Disease Prevention and Health Promotion, CDC; ^2^Division of Congenital and Developmental Disorders, National Center on Birth Defects and Developmental Disabilities.; Florida Department of Health; Georgia Department of Public Health; Virginia Department of Health; South Carolina Department of Health and Environmental Control; Georgia Department of Public Health; Louisiana Department of Health; Louisiana State University; Kentucky Department for Public Health; Alabama Department of Public Health; Maryland Department of Health; Maryland Department of Health; Florida Department of Health; Mississippi State Department of Health; Alabama Department of Public Health; South Carolina Department of Health and Environmental Control; Minnesota Department of Health; Puerto Rico Department of Health; West Virginia Department of Health and Human Resources; Ohio Department of Health; Puerto Rico Department of Health; Connecticut Department of Public Health.

## Abstract

Ensuring access to and promoting use of effective contraception have been identified as important strategies for preventing unintended pregnancy (*1*). The importance of ensuring resources to prevent unintended pregnancy in the context of public health emergencies was highlighted during the 2016 Zika virus outbreak when Zika virus infection during pregnancy was identified as a cause of serious birth defects (*2*). Accordingly, CDC outlined strategies for state, local, and territorial jurisdictions to consider implementing to ensure access to contraception (*3*). To update previously published contraceptive use estimates* among women at risk for unintended pregnancy^†^ and to estimate the number of women with ongoing or potential need for contraceptive services,^§^^,^^¶^ data on contraceptive use were collected during September–December 2016 through the Behavioral Risk Factor Surveillance System (BRFSS). Results from 21 jurisdictions indicated that most women aged 18–49 years were at risk for unintended pregnancy (range across jurisdictions = 57.4%–76.8%). Estimates of the number of women with ongoing or potential need for contraceptive services ranged from 368 to 617 per 1,000 women aged 18–49 years. The percentage of women at risk for unintended pregnancy using a most or moderately effective contraceptive method** ranged from 26.1% to 65.7%. Jurisdictions can use this information to estimate the number of women who might seek contraceptive services and to plan and evaluate efforts to increase contraceptive use. This information is particularly important in the context of public health emergencies, such as the recent Zika virus outbreak, which have been associated with increased risk for adverse maternal-infant outcomes (*2*,*4*–*6*) and have highlighted the importance of providing women and their partners with resources to prevent unintended pregnancy.

BRFSS is a cross-sectional jurisdiction-specific, random-digit–dialed, telephone survey that collects data on risk behaviors and preventive health practices among adult respondents living in the 50 states, the District of Columbia, Puerto Rico, Guam, and U.S. Virgin Islands.^††^ This report includes data from 21 jurisdictions^§§^ that implemented the optional family planning module on self-reported contraceptive use during September–December 2016.^¶¶^ Individual contraceptive methods from this module were classified according to first-year typical use failure rates as most effective (≤1% failure), moderately effective (>1%–10% failure), or less effective (>10% failure).*** Women reporting more than one contraceptive method were classified according to the most effective method they reported using.

Weighted estimates and 95% confidence intervals were calculated to determine the proportion of women aged 18–49 years at risk for unintended pregnancy (defined as those who reported they were sexually active with a male partner, but did not report that they were currently pregnant or seeking pregnancy, that they would not mind being pregnant, or that they had a hysterectomy). In addition, numbers and rates (total number and number per 1,000 women aged 18–49 years) and corresponding 95% confidence intervals were calculated for women with ongoing or potential need for contraceptive services (defined as those at risk for unintended pregnancy who were not using permanent contraceptive methods [female sterilization or report of male partner vasectomy]). Estimates also were calculated to describe the proportion of women at risk for unintended pregnancy using contraception by effectiveness category (most effective, including permanent methods and long-acting reversible contraception [LARC]; moderately effective; less effective; and no method). Estimates for using either a less effective method or no method were further stratified by age group (18–24, 25–34, 35–44, and 45–49 years). Women at risk for unintended pregnancy who did not specify the type of contraception they used or reported “other” methods (4.8%)^†††^ were excluded from estimates of contraceptive use by method effectiveness and from estimates of the number of women with ongoing or potential need for contraceptive services. Estimates that did not meet reliability standards established for BRFSS were suppressed.^§§§^

Among the 21 jurisdictions, the proportion of women aged 18–49 years at risk for unintended pregnancy ranged from 57.4% (Texas) to 76.8% (Minnesota) (Table 1). Jurisdictions with the fewest numbers of women with ongoing or potential need for contraceptive services included Guam, Kansas, Puerto Rico, and West Virginia; jurisdictions with the highest numbers included California, Florida, Illinois, and Texas. Estimates of the number of women with ongoing or potential need for contraceptive services per 1,000 women aged 18–49 years ranged from 368 in Puerto Rico to 617 in Maryland. Among women at risk for unintended pregnancy, the proportion using either a most or moderately effective contraceptive method ranged from 26.1% (Guam) to 65.7% (West Virginia) (Table 2); among 11 jurisdictions with reliable estimates for LARC, use ranged from 5.5% (Kansas) to 17.0% (Maryland). Among 18 jurisdictions with reliable estimates, the percentage of women at risk for unintended pregnancy using a less effective method of contraception ranged from 11.1% (Illinois) to 47.7% (Arizona), and among 19 jurisdictions, the percentage not using any method of contraception ranged from 16.5% (Virginia) to 63.0% (Guam) (Table 3). Across age-stratified estimates, the percentage using either a less effective method or no method ranged from 25.9% (women aged 35–44 years in South Carolina) to 79.9% (women aged 18–24 years in California) (Supplementary Table, https://stacks.cdc.gov/view/cdc/57915).

**TABLE 1:** Percentage of women aged 18–49 years at risk for unintended pregnancy* and numbers of women with ongoing or potential need for contraceptive services,^†^^,^^§^ by jurisdiction — Behavioral Risk Factor Surveillance System, 21 jurisdictions, September–December, 2016

Jurisdiction	Total no. of women aged 18–49 years^¶^	% of women aged 18–49 years at risk for unintended pregnancy (95% CI)	Women with ongoing or potential need for contraceptive services	
			No. (95% CI)^¶^	No. per 1,000 aged 18–49 years (95% CI)
Alabama	1,022,400	64.6 (56.9–71.6)	418,200 (342,500–498,400)	409 (335–487)
Arizona	1,400,300	57.9 (42.9–71.5)	683,400 (487,400–882,200)	488 (348–630)
California	8,585,800	67.6 (60.3–74.1)	4,464,500 (3,817,200–5,104,000)	520 (445–594)
Connecticut	737,700	67.2 (51.5–79.9)	378,800 (283,900–472,400)	514 (385–640)
Florida	4,027,500	59.9 (53.4–66.1)	1,803,900 (1,566,300–2,047,500)	448 (389–508)
Georgia	2,252,800	62.5 (50.3–73.2)	1,089,400 (828,400–1,354,400)	484 (368–601)
Illinois	2,745,600	74.1 (63.9–82.1)	1,675,800 (1,380,200–1,944,600)	610 (503–708)
Kansas	588,900	71.9 (66.8–76.5)	297,100 (262,900–331,300)	505 (446–563)
Kentucky	913,400	71.8 (66.8–76.3)	447,900 (397,400–498,600)	490 (435–546)
Louisiana	997,700	62.1 (44.0–77.3)	387,800 (227,600–576,400)	389 (228–578)
Maryland	1,299,200	75.8 (69.3–81.3)	801,200 (707,500–888,600)	617 (545–684)
Minnesota	1,126,900	76.8 (70.3–82.3)	596,800 (502,200–689,700)	530 (446–612)
New Jersey	1,862,500	76.6 (65.4–85.0)	1,142,400 (922,100–1,340,300)	613 (495–720)
Ohio	2,359,500	61.5 (52.9–69.4)	1,105,200 (907,800–1,306,900)	468 (385–554)
Oklahoma	805,100	65.8 (58.5–72.5)	376,800 (318,000–436,600)	468 (395–542)
South Carolina	1,021,100	70.3 (62.7–76.9)	548,300 (461,100–633,400)	537 (452–620)
Texas	6,011,100	57.4 (47.4–66.9)	2,435,800 (1,888,700–3,025,200)	405 (314–503)
Virginia	1,813,800	71.6 (64.1–78.1)	938,500 (799,900–1,075,600)	517 (441–593)
West Virginia	360,400	67.6 (61.4–73.3)	158,200 (136,700–180,300)	439 (379–500)
Guam	35,200	70.3 (59.2–79.4)	20,800 (16,500–24,700)	591 (469–702)
Puerto Rico	795,700	63.7 (58.8–68.4)	292,900 (255,600–332,200)	368 (321–417)

**Abbreviation:** CI = confidence interval.

* Women were considered at risk for unintended pregnancy unless they reported that they were not sexually active with a male partner, that they were currently pregnant or seeking pregnancy, that they would not mind being pregnant, or that they had a hysterectomy.

^†^ Women with ongoing or potential need for contraceptive services were defined as those women considered at risk for unintended pregnancy who were not using permanent contraceptive methods (female sterilization or report of male partner vasectomy).

^§^The number of women with ongoing or potential need for contraceptive services can be used to predict how many women might seek services; this measure does not represent unmet need for contraception because many of these women might already be using some method of contraception: https://www.guttmacher.org/sites/default/files/report_pdf/contraceptive-needs-and-services-2014_1.pdf.

^¶^ Numbers are rounded to the nearest 100.

**TABLE 2:** Percentage of women aged 18–49 years at risk for unintended pregnancy* using most**^†^** or moderately effective**^§^** contraceptive methods, by jurisdiction — Behavioral Risk Factor Surveillance System, 21 jurisdictions, September–December, 2016

Jurisdiction	Total	Most effective		Moderately effective
	Most or moderately effective	Sterilization	Long-acting reversible (LARC)	
	% (95% CI)	% (95% CI)	% (95% CI)	% (95% CI)
Alabama	63.8 (54.0–72.5)	35.7 (26.6–46.0)	—^¶^	19.1 (12.5–28.2)
Arizona	39.1 (25.1–55.1)	—	—	—
California	51.5 (42.0–60.9)	22.5 (16.5–29.9)	11.7 (7.5–17.8)	17.2 (12.4–23.4)
Connecticut	55.4 (44.5–65.9)	21.9 (15.0–31.0)	9.4 (5.5–15.7)	24.1 (14.6–37.0)
Florida	48.6 (42.0–55.2)	22.9 (17.3–29.6)	9.7 (6.5–14.4)	16.0 (12.0–21.0)
Georgia	51.5 (36.9–65.8)	22.3 (12.0–37.7)	—	—
Illinois	62.4 (50.5–73.0)	16.8 (10.0–26.7)	—	33.3 (21.0–48.3)
Kansas	60.9 (53.7–67.7)	28.9 (23.0–35.8)	5.5 (3.3–9.1)	26.4 (20.6–33.2)
Kentucky	60.1 (53.4–66.5)	31.3 (25.1–38.2)	6.6 (4.1–10.6)	22.2 (17.0–28.4)
Louisiana	56.9 (32.1–78.7)	35.0 (18.7–55.8)	—	—
Maryland	62.3 (53.8–70.1)	17.6 (12.7–23.9)	17.0 (11.0–25.4)	27.7 (19.5–37.6)
Minnesota	60.2 (50.2–69.4)	29.9 (21.3–40.2)	11.8 (6.6–20.2)	18.5 (11.1–29.2)
New Jersey	50.8 (37.2–64.2)	16.3 (10.5–24.5)	—	—
Ohio	45.4 (35.7–55.3)	22.9 (16.6–30.6)	7.6 (4.4–13.0)	14.8 (10.0–21.5)
Oklahoma	62.5 (53.0–71.1)	28.2 (21.3–36.3)	—	27.0 (19.5–36.0)
South Carolina	61.5 (50.3–71.7)	22.8 (15.5–32.3)	10.5 (5.8–18.3)	28.2 (19.4–39.2)
Texas	53.0 (40.7–65.1)	27.3 (17.7–39.5)	—	20.5 (12.8–31.1)
Virginia	60.8 (51.9–68.9)	26.8 (20.0–35.0)	13.3 (7.8–21.7)	20.7 (14.8–28.0)
West Virginia	65.7 (58.9–72.0)	34.4 (27.8–41.6)	11.0 (6.5–17.9)	20.4 (15.0–27.0)
Guam	26.1 (15.2–41.0)	—	—	—
Puerto Rico	49.8 (43.6–55.9)	41.6 (35.7–47.8)	—	6.8 (4.1–11.1)

**Abbreviation:** CI = confidence interval

* Women were considered at risk for unintended pregnancy unless they reported that they were not sexually active with a male partner, that they were currently pregnant or seeking pregnancy, that they would not mind being pregnant, or that they had a hysterectomy.

^†^ Most effective contraceptive methods included permanent methods (female sterilization or report of male partner vasectomy) and long-acting reversible contraception (LARC, including intrauterine devices [IUDs] and contraceptive implants); most effective methods have a ≤1% failure rate during the first year of typical use. Sources: Trussell J. Contraceptive failure in the United States. Contraception 2011;83:397–404. Sundaram A, Vaughan B, Kost K, et al. Contraceptive failure in the United States: estimates from the 2006–2010 National Survey of Family Growth. Perspect Sex Reprod Health 2017;49:7–16.

^§^ Moderately effective contraceptive methods included contraceptive injectables, contraceptive pills, transdermal contraceptive patches, and vaginal rings; moderately effective methods have a >1%–10% failure rate with typical use. Sources: Trussell J. Contraceptive failure in the United States. Contraception 2011;83:397–404. Sundaram A, Vaughan B, Kost K, et al. Contraceptive failure in the United States: estimates from the 2006–2010 National Survey of Family Growth. Perspect Sex Reprod Health 2017;49:7–16.

^¶^ Estimate is unreliable (relative standard error >30% or denominator <50).

**TABLE 3:** Percentage of women aged 18–49 years at risk for unintended pregnancy* using less effective**^†^** contraceptive methods or no method, by jurisdiction — Behavioral Risk Factor Surveillance System, 21 jurisdictions, September–December, 2016

Jurisdiction	Total	Less effective method	No method
	Less effective or no method		
	% (95% CI)	% (95% CI)	% (95% CI)
Alabama	36.2 (27.5–46.0)	13.6 (8.3–21.6)	22.6 (15.6–31.6)
Arizona	60.9 (44.9–74.9)	47.7 (31.0–65.0)	—^§^
California	48.5 (39.1–58.0)	31.6 (21.9–43.2)	16.9 (12.3–22.9)
Connecticut	44.6 (34.1–55.5)	20.4 (13.0–30.6)	24.1 (16.4–34.1)
Florida	51.4 (44.8–58.0)	14.1 (10.2–19.3)	37.3 (31.2–43.9)
Georgia	48.5 (34.2–63.1)	—	34.1 (21.9–48.8)
Illinois	37.6 (27.0–49.5)	11.1 (6.2–19.1)	26.4 (18.0–37.0)
Kansas	39.1 (32.3–46.3)	14.5 (10.4–19.8)	24.6 (18.7–31.7)
Kentucky	39.9 (33.5–46.6)	20.0 (15.0–26.1)	19.9 (15.5–25.2)
Louisiana	43.1 (21.3–67.9)	—	—
Maryland	37.7 (29.9–46.2)	18.8 (13.3–26.0)	18.9 (13.4–25.8)
Minnesota	39.8 (30.6–49.8)	13.1 (8.1–20.6)	26.7 (19.0–36.1)
New Jersey	49.2 (35.8–62.8)	18.3 (10.7–29.6)	30.9 (21.3–42.5)
Ohio	54.6 (44.7–64.3)	22.2 (13.0–35.2)	32.5 (23.5–43.0)
Oklahoma	37.5 (28.9–47.0)	11.8 (7.9–17.3)	25.7 (17.8–35.6)
South Carolina	38.5 (28.3–49.7)	11.3 (7.7–16.3)	27.2 (17.6–39.5)
Texas	47.0 (34.9–59.3)	16.0 (9.8–24.9)	31.0 (19.7–45.0)
Virginia	39.2 (31.1–48.1)	22.7 (15.6–31.8)	16.5 (11.9–22.4)
West Virginia	34.3 (28.0–41.1)	11.9 (8.4–16.7)	22.3 (17.2–28.5)
Guam	74.0 (59.0–84.8)	—	63.0 (47.7–76.0)
Puerto Rico	50.2 (44.1–56.4)	20.1 (15.5–25.6)	30.2 (24.8–36.1)

**Abbreviation:** CI = confidence interval.

* Women were considered at risk for unintended pregnancy unless they reported that they were not sexually active with a male partner, that they were currently pregnant or seeking pregnancy, that they would not mind being pregnant, or that they had a hysterectomy.

^†^ Less effective contraceptive methods included diaphragms, condoms (male or female), withdrawal, cervical caps, sponges, spermicides, fertility-awareness based methods, and emergency contraception; less effective methods have a >10% failure rate during the first year of typical use. Sources: Trussell J. Contraceptive failure in the United States. Contraception 2011;83:397–404. Sundaram A, Vaughan B, Kost K, et al. Contraceptive failure in the United States: estimates from the 2006–2010 National Survey of Family Growth. Perspect Sex Reprod Health 2017;49:7–16.

^§^ Estimate is unreliable (relative standard error >30% or denominator <50).

## Discussion

Across the 21 jurisdictions, the number of women with ongoing or potential need for contraceptive services per 1,000 women aged 18–49 years ranged from 368 to 617 and exceeded 4 million in total in the jurisdiction with the highest number of women with ongoing or potential need for contraceptive services. The proportion of women at risk for unintended pregnancy using a most or moderately effective method of contraception ranged from 26.1% to 65.7%. The proportion using no contraception ranged from 16.5% to 63.0%. These data can be used for jurisdictional planning and are particularly important in the context of public health emergencies associated with increased risk for adverse maternal-infant outcomes that heighten the need to provide women and their partners with resources to prevent unintended pregnancy.

The data for this report were collected because of concerns about Zika virus–related adverse pregnancy and birth outcomes; however, the findings have broader implications. Several types of public health emergencies, such as natural disasters, including hurricanes, have been associated with adverse maternal-infant outcomes, along with disruptions in women’s abilities to access contraception and interruptions in method use (*4*,*5*). Similarly, given the ongoing opioid crisis and high proportion of unintended pregnancies among women who misuse opioids (*6*), ensuring access to contraception and preconception care among these women is an important strategy for reducing the incidence of neonatal abstinence syndrome (*6*). Moreover, ensuring access to effective contraception is important in general for supporting women and their partners in planning their pregnancies and is also cost-saving (*7*), particularly during public health emergencies such as the Zika virus outbreak where costs associated with long-term care of children with adverse birth outcomes are high (*8*).

Jurisdiction-level data are important because of the substantial variation among jurisdictions in unintended pregnancy rates (*9*). Although a number of sociodemographic factors contribute to this variation, implementation of programs and policies that increase access to contraception, including the most effective methods, also varies considerably among jurisdictions.^¶¶¶^ During the Zika virus outbreak response, CDC worked with jurisdictional partners to implement strategies to promote increased access to contraception (*3*). Frequently adopted strategies included maintaining sustainable partnerships among insurers, manufacturers, and state agencies; reimbursing for the full range of contraceptive services; maintaining continuous stocking and supply of devices in a wide range of service facilities; and training providers on current insertion and removal techniques for the most effective methods. Although developed during the Zika virus response, these strategies apply broadly to all situations in which women and their partners need access to resources to prevent unintended pregnancy.

This report provides data both for estimating the number of women who might seek services and for evaluating the impact of policies and programs. Understanding how many women need contraceptive services and where the need is greatest can aid in planning health care delivery.**** In addition, the proportion of women at risk for unintended pregnancy using a most or moderately effective contraceptive method is an established indicator of quality family planning service provision^††††^ and a *Healthy People 2020* objective.^§§§§^ This indicator is critical for evaluating the success of implementation strategies and population-level impact (*1*). Conversely, variation in prevalence of use of less effective contraceptive methods or no method, as documented in this report by age group, can be used to identify the need for targeted implementation of strategies, such as provision of youth-friendly services (*3*).

The findings in this report are subject to at least five limitations. First, information on contraceptive use was self-reported and might be subject to recall or social desirability bias. Second, because data for this report were collected over a 4-month period versus an entire year, small sample sizes limited the precision of estimates. Third, it was not possible to determine whether those reporting unspecified methods were using permanent or reversible methods. Estimates of the number of women with ongoing or potential need for contraceptive services therefore excluded these women and might have underestimated the number who might seek services; conversely, these estimates included women using LARC, who might only need services every 3–10 years depending on the type of LARC (*10*). Fourth, this report includes data from only 21 jurisdictions and is not representative of other jurisdictions; however, it highlights the need for ongoing collection of jurisdiction-level data for all U.S. jurisdictions. Finally, nonresponse bias remains a possibility, although the weighting methodology used by BRFSS adjusts for nonresponse bias.

Ensuring access to effective contraception is an important strategy for preventing unintended pregnancy and can be particularly important in the context of certain public health responses. During the Zika virus outbreak, contraception served as a medical countermeasure to prevent Zika virus-affected pregnancies and is similarly important in other contexts where risk for adverse maternal-infant outcomes is increased. The data in this report can be applied in nonemergency settings to help jurisdictions estimate the number of women who might seek contraceptive services and to plan and evaluate implementation strategies.

**Summary**

**What is already known about this topic?**

Ensuring access to contraception is an effective strategy for preventing unintended pregnancy and associated negative maternal-infant outcomes.

**What is added by this report?**

Data from 21 jurisdictions collected during a 4-month period indicated the number of women with ongoing or potential need for contraceptive services per 1,000 women aged 18–49 years. ranged from 368 to 617. The proportion at risk for unintended pregnancy using a most or moderately effective contraceptive method ranged from 57.4% to 76.8%. The proportion using no contraception ranged from 16.5% to 63.0%.

**What are the implications for public health practice?**

The recent Zika virus outbreak highlighted the need for contraception data in the context of public health responses associated with adverse maternal-infant outcomes. These data can inform delivery of contraceptive services and evaluation of implementation strategies to increase access to contraception.

## Footnotes

* State-based estimates of contraceptive use during the Zika response were from 2011–2013. https://www.cdc.gov/mmwr/volumes/65/wr/mm6530e2.htm.

† Women were considered at risk for unintended pregnancy unless they reported that they were not sexually active with a male partner, that they were currently pregnant or seeking pregnancy, that they would not mind being pregnant, or that they had a hysterectomy.

§ Women with ongoing or potential need for contraceptive services were defined as those women considered at risk for unintended pregnancy who were not using permanent contraceptive methods (female sterilization or report of male partner vasectomy).

†† https://www.cdc.gov/brfss/data_documentation/index.htm.

§§ Includes Alabama, Arizona, California, Connecticut, Florida, Georgia, Illinois, Kansas, Kentucky, Louisiana, Maryland, Minnesota, New Jersey, Ohio, Oklahoma, South Carolina, Texas, Virginia, West Virginia, Guam, and Puerto Rico. Data collected for Mississippi are not included in this report because they did not meet BRFSS reliability standards (denominators ≥50 respondents and a relative standard error ≤30%) with respect to reporting the number of women with ongoing or potential need for contraceptive services, or the proportion of women at risk for unintended pregnancy by method type.

¶¶ Questions implemented followed those implemented in 2017 with Module 17: Preconception Health/Family Planning. https://www.cdc.gov/brfss/questionnaires/pdf-ques/2017_BRFSS_Pub_Ques_508_tagged.pdf.

*** Most effective contraceptive methods included permanent contraceptive methods (female sterilization or report of male partner vasectomy) and long-acting reversible contraception (LARC, including intrauterine devices [IUDs] and contraceptive implants). Moderately effective contraceptive methods included contraceptive injectables, contraceptive pills, contraceptive patches, and vaginal rings. Less effective contraceptive methods included diaphragms, condoms (male or female), withdrawal, cervical caps, sponges, spermicides, fertility-awareness based methods, and emergency contraception.

††† Write-in responses were not available for women responding “other,” and previous evaluation of BRFSS contraceptive use data indicates these methods are a mix of permanent and reversible methods of all effectiveness levels. https://www.cdc.gov/mmwr/volumes/65/wr/mm6530e2.htm.

§§§ Reliability standards for BRFSS require suppression of estimates with an unweighted denominator of <50 respondents or a relative standard error >30%.

¶¶¶ Examples of programs and policies that vary by state include participation in the Association of State and Territorial Health Officials’ state learning community for improving access http://www.astho.org/Programs/Maternal-and-Child-Health/Increasing-Access-to-Contraception/ and Medicaid family planning eligibility expansions https://www.guttmacher.org/state-policy/explore/medicaid-family-planning-eligibility-expansions.

**** https://www.guttmacher.org/sites/default/files/report_pdf/contraceptive-needs-and-services-2014_1.pdf and https://thenationalcampaign.org/deserts.

†††† https://www.hhs.gov/opa/performance-measures/most-or-moderately-effective-contraceptive-methods/index.html.

§§§§ https://www.healthypeople.gov/2020/topics-objectives/topic/family-planning/objectives; FP-16.
